# Vitamin D supplementation as a potential cause of U-shaped associations between vitamin D levels and negative health outcomes: a decision tree analysis for risk of frailty

**DOI:** 10.1186/s12877-017-0631-0

**Published:** 2017-10-16

**Authors:** Gotaro Kojima, Steve Iliffe, Marianne Tanabe

**Affiliations:** 1 Department of Primary Care and Population Health, University College London (Royal Free Campus), Rowland Hill Street, London, NW3 2PF UK; 2 Veterans Affairs Pacific Islands Health Care System, Honolulu, Hawaii USA

**Keywords:** Frailty, Vitamin D, Vitamin D deficiency, Vitamin D supplementation, Nursing home

## Abstract

**Background:**

A recent controversy in vitamin D research is a “U-shaped association”, with elevated disease risks at both high and low 25-hydroxyvitamin D (25 (OH) D) levels.

**Methods:**

This is a cross-sectional study of 238 male nursing home veterans in Hawaii. Classification and regression tree (CART) analysis identified groups based on 25 (OH) D and vitamin D supplementation for frailty risk. Characteristics were examined and compared across the groups using logistic regression and receiver operating characteristic (ROC) curve analyses.

**Results:**

CART analysis identified three distinct groups: vitamin D supplement users (*n* = 86), non-users with low vitamin D (*n* = 55), and non-users with high vitamin D (*n* = 97). Supplement users were the most frail, but had high mean 25 (OH) D of 26.6 ng/mL, which was compatible with 27.1 ng/mL in non-users with high vitamin D, while mean 25 (OH) D of non-users with low vitamin D was 11.7 ng/mL. Supplement users and non-users with low vitamin D were significantly more likely to be frail (odds ratio (OR) = 9.90, 95% CI = 2.18–44.86, *p* = 0.003; OR = 4.28, 95% CI = 1.44–12.68, *p* = 0.009, respectively), compared with non-users with low vitamin D. ROC curve analysis showed the three groups significantly predicted frailty (area under the curve = 0.73), with sensitivity of 64.4% and specificity of 76.7%, while 25 (OH) D did not predict frailty.

**Conclusions:**

In these nursing home veterans, vitamin D supplement users were the most frail but with high 25 (OH) D. This can potentially be a cause of U-shaped associations between vitamin D levels and negative health outcomes.

**Electronic supplementary material:**

The online version of this article (doi: 10.1186/s12877-017-0631-0) contains supplementary material, which is available to authorized users.

## Background

Vitamin D deficiency is prevalent worldwide and has been recognized as a public health problem [1]. Low vitamin D has been extensively studied and shown to be associated with various negative health outcomes, such as osteoporosis, fracture, muscle weakness, falls, autoimmune diseases, and cardiovascular diseases [2]. Optimizing deficient vitamin D levels with supplementation can result in mostly favorable results, although definitive evidence from well-designed randomized controlled clinical trials is lacking [3]. In light of the accumulating evidence on the beneficial effects of vitamin D supplementation and its relatively safe side-effect profile, vitamin D supplementation has been recommended by multiple authorities and guidelines [4–6].

One of the recent controversies in vitamin D research is a “U-shaped association”, [7–9] in which both high and low levels of vitamin D are associated with elevated disease risks [7]. Regarding all-cause mortality, most observational population-based studies have shown that low vitamin D predicted premature death, which is supported by recent meta-analyses [10–13]. However, there were a considerable number of studies that showed U-shaped or reverse J-shaped associations between vitamin D and mortality [14–20]. One of the meta-analysis studies showed the possibility that mortality risk may become higher again at a serum 25-hydroxyvitamin D (25 (OH) D) level of 112.5 nmol/l or more [12]. The association between high vitamin D and high mortality appears counterintuitive and the underlying mechanisms are unknown. Criticisms of this paradoxical finding were that it was due to chance, [7] a lack of adequate adjustment, [8] an analytical bias due to serum vitamin D assays, [7] a small number of the highest serum vitamin D group, [18] or vitamin D intoxication [21].

Use of vitamin D supplementation may be a cause of increased disease risks at high vitamin D levels and can create U-shaped associations [22]. The Newcastle 85+ Study prospectively followed 775 men and women aged 85 or older over 6 years and examined all-cause mortality according to baseline 25 (OH) D levels [13]. Compared with the middle 25 (OH) D group, the highest and lowest 25 (OH) D groups had non-significantly increased mortality risks (adjusted hazard ratio [HR] = 1.25, 95% confidence interval [CI] = 0.97–1.63; HR = 1.10, 95% CI = 0.85–1.42, respectively). After excluding 150 vitamin D supplement users, the mortality risk for the highest 25 (OH) D group decreased by 16% (adjusted HR = 1.05, 95% CI = 0.73–1.53) whereas the mortality risk for the lowest 25 (OH) D group increased (adjusted HR = 1.22, 95% CI = 0.93–1.60) [13]. It is speculated that the supplement users were at high risk with high 25 (OH) D, likely due to the supplements, which led to the increased mortality risk of the highest 25 (OH) D group. Another meta-analysis study showed that the mortality risk for low 25 (OH) D was significantly lower among studies with prevalence of vitamin D supplement use of more than 10% compared with studies with less than 10% (p for meta-regression analysis <0.05), [11] which suggests that vitamin D supplementation attenuated inverse association between vitamin D and mortality, possibly by increasing 25 (OH) D of high-mortality-risk participants.

In order to further investigate how vitamin D supplementation affects associations between vitamin D status and health outcomes, we explored use of vitamin D supplementation and serum 25 (OH) D in relation to frailty, using a cohort of frail nursing home residents [23] among whom prevalence of both vitamin D supplement use and vitamin D deficiency are high. Our hypothesis was that those on a vitamin D supplement were highly frail even though serum vitamin D levels were elevated by supplements. They could be included in “high” vitamin D groups along with healthier vitamin D sufficient non-supplement users, leading to the seemingly paradoxically high risks of various health outcomes in those with high vitamin D.

## Methods

### Study design, setting, and population

This cross-sectional study was conducted at a Veterans Affairs nursing home in Honolulu, Hawaii, providing rehabilitation, skilled-nursing care, intermediate care, respite care, and hospice/palliative care for veterans. The study participants were all male veterans admitted to the study nursing home except for those admitted for hospice/palliative care, from 1st of January 2011 to 31st of December 2012. The data were anonymised and collected by a retrospective chart review. This study was approved by the Institutional Review Boards of Veterans Affairs Pacific Islands Health Care System. The study design, setting, and population have been described in detail elsewhere [24, 25].

### Predictor variables - vitamin D supplementation and vitamin D level

Serum total 25 (OH) D was measured for all veterans on nursing home admission as a part of initial assessment. The information on vitamin D supplementation was obtained from transfer summaries, discharge summaries, outpatient clinic notes, or history taken from veterans or family. Various dosage, frequency, and duration were observed for vitamin D supplementation, from one multivitamin tab a day to 50,000 IU of ergocalciferol three times weekly. Use of vitamin D supplementation was calculated as a mean daily dosage of any vitamin D supplement over the previous 1 month and categorized into four groups: no supplement, 1–400 IU/day, 401–800 IU/day, or ≥801 IU/day.

### Outcome variable - frailty

Frailty has been described as a state of reduced physiological reserve due to age related accumulation of multisystem impairments [26]. As people become frailer, they are more predisposed to increased risks of various adverse health outcomes, including falls, fractures, hospitalization, nursing home placement, disability, poor quality of life, and dementia [27–34]. Therefore frailty was considered as a good surrogate marker of biological aging [35]. In this study, frailty was measured by using a deficit accumulation model of the Frailty Index (FI) [35] FI is calculated from a variety of health deficits that usually include symptoms, signs, comorbidities, and disabilities that are biologically sensible, accumulate with age, do not saturate too early, and cover a range of systems [36]. Although FI does not require the same number of deficits or the same set of deficits, it is recommended to include at least 30 deficits [36]. We constructed FI based on 34 deficits including 12 chronic diseases, 9 psychological symptoms, 6 functional disabilities, 3 gait/fall-related problems, 2 cognitive symptoms, 1 obesity, and 1 pain symptom. (see the Additional file 1 for detail) [36, 37]. Although FI is a continuous score and not meant to be dichotomized, we used an empirical cut-point to define frailty as FI > =0.25 [38].

### Covariates

Demographic data collected on admission were age, body mass index, ethnicity (White, Asian/Pacific Islander, or other), education, smoking, alcohol, place where veterans came from (home, acute care hospitals, or other nursing homes), season and reason for admission (rehabilitation, non-rehabilitation skilled-nursing care, intermediate care, or respite care).

### Statistical analysis

The classification and regression tree (CART) analysis [39] is a non-parametric classification technique that can deal with multiple predictors of both continuous and categorical data. It builds a decision tree by recursive partitioning to best explain the risk estimate of the dependent variable. This method has often been used for data mining and it was considered to be appropriate for the exploratory nature of this study. We used this method to split the cohort based on two variables: use of vitamin D supplementation and 25 (OH) D, into progressively smaller and more homogeneous subgroups with highest discriminative ability of identifying frailty risk. The minimum number of cases in a node was set at 23, 1/10 of the entire cohort, and the minimum change in improvement was set at 0.001. Ten-fold cross-validation was performed. The subgroups by the CART analysis were compared for the characteristics using a one-way analysis of variance (ANOVA) for continuous variables and a chi-square test for categorical variables. Correlation coefficient was examined using Spearman’s rho between 25 (OH) D and the FI in the entire cohort as well as supplement users and non-users. Relative likelihood of frailty of each CART group compared with the entire cohort was calculated. Univariate logistic regression models were used to examine risk of frailty for the CART groups and other characteristics. The CART groups were further examined for independent risk of frailty using a multivariate logistic regression model adjusted for variables which were significant in the univariate logistic regression models. Frailty risk discrimination by the CART analysis was assessed using the receiver operating characteristic (ROC) curve analysis and the area under the ROC curve (AUC), and was compared with that by 25 (OH) D as a continuous variable.

Among the supplement users, the characteristics were compared using a one-way ANOVA for continuous variables and a chi-square test for categorical variables according to the dosage of the supplement.

All statistical analyses were conducted using IBM SPSS Statistics (version 20, IBM Corporation, Armonk, NY, USA), and two-sided *p* value of <0.05 was considered statistically significant.

## Results

There were a total of 302 male veterans admitted to the study nursing home during 2011 and 2012. Of those, 61 veterans who did not have 25 (OH) D measured within 7 days of admission and 3 veterans who missed more than 30% of deficits for constructing FI were excluded, leaving 238 veterans (78.8%) as a final sample. There were no significant differences in mean age, mean 25 (OH) D level, BMI, smoking, alcohol use and FI between those included (*n* = 238) and those excluded (*n* = 64). Those excluded had slightly but significantly longer duration of education compared with those included (13.7 v 13.0 years).

### Decision tree analysis

**Fig. 1:**
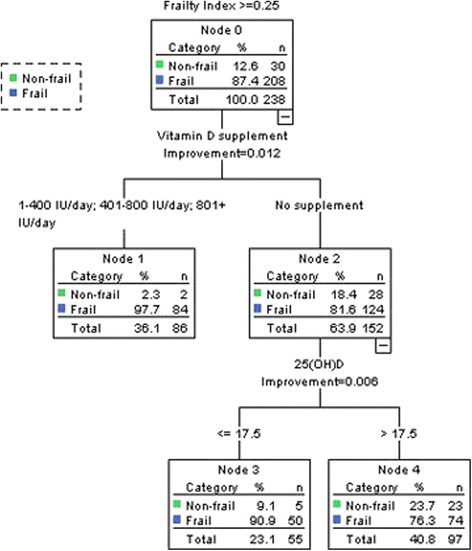
A decision tree by classification and regression tree analysis based on use of vitamin D supplement and 25-hydroxyvitamin D (25 (OH) D) among 238 male veterans in a nursing home. 25 (OH) D: 25-hydroxyvitamin D

Figure 1 displays a decision tree of the CART analysis. The cohort was initially divided into two groups: those on vitamin D supplement of any dosage (1–400 IU/day, 401–800 IU/day, or ≥801 IU/day, vitamin D supplement users, *n* = 86) and those without vitamin D supplement (non-users, *n* = 152). Non-users were further divided based on 25 (OH) D with a cut-off point of 17.5 ng/mL into two groups: non-users with low vitamin D level (*n* = 55) and non-users with high vitamin D level (*n* = 97). The cross-validation showed the same risk estimate. As a supplementary analysis the CART analysis was repeated using the FI as a continuous outcome variable, which showed very similar results (data not shown).

### Cohort characteristics

Table 1 presents demographic characteristics of the entire cohort (*N* = 238). The mean age was 73.4 years. The mean Frailty Index (FI) was 0.39 and 87.4% (*n* = 208) had frailty (FI > =0.25). The mean 25 (OH) D was 23.4 ng/mL, ranging 4–53 ng/mL, and 37.4% (*n* = 89) had 25 (OH) D < 20 ng/mL.

**Table 1:** Characteristics according to three groups based on vitamin D level and supplement use

	Entire cohort	Supplement users	Non-users (*n* = 152)		
	*N* = 238	*n* = 86 (36.1%)	Low vitamin D (<18 ng/mL) *n* = 55 (23.1%)	High vitamin D (> = 18 ng/mL) *n* = 97 (40.8%)	*p* value
Frailty Index	0.39 ± 0.13	0.43 ± 0.11	0.41 ± 0.13	0.35 ± 0.13	<0.001
Frailty (FI > =0.25)	208 (87.4%)	84 (97.7%)	50 (90.9%)	74 (76.3%)	<0.001
25 (OH) D (ng/mL)					
Mean	23.4 ± 9.8	26.6 ± 9.3	11.7 ± 3.8	27.1 ± 7.0	<0.001
Range	4–53	6–52	4–17	18–53	
< 20 ng/mL	89 (37.4%)	19 (22.1%)	55 (100.0%)	15 (15.5%)	<0.001
Age	73.4 ± 13.1	79.0 ± 11.7	67.4 ± 12.1	71.9 ± 13.0	<0.001
Body mass index	26.5 ± 6.7	25.1 ± 6.0	29.5 ± 8.5	26.0 ± 5.5	<0.001
Education (year)	13.0 ± 2.3	13.0 ± 2.4	13.0 ± 3.0	13.0 ± 2.3	1.00
Ethnicity					
White	117 (49.2%)	41 (47.7%)	21 (38.2%)	55 (56.7%)	0.03
Asian/Pacific Islander	102 (42.9%)	41 (47.7%)	30 (54.5%)	31 (32.0%)	
Others	18 (7.6%)	3 (3.5%)	4 (7.3%)	11 (11.3%)	
Smoking					
Never	80 (33.6%)	27 (31.4%)	13 (23.6%)	40 (41.2%)	0.10
Past	120 (50.4%)	49 (57.0%)	30 (54.5%)	41 (42.3%)	
Current	38 (16.0%)	10 (11.6%)	12 (21.8%)	16 (16.5%)	
Alcohol					
Never	100 (42.0%)	39 (45.3%)	20 (36.4%)	41 (42.3%)	0.73
Past	83 (34.9%)	30 (34.9%)	22 (40.0%)	31 (32.0%)	
Current	55 (23.1%)	17 (19.8%)	13 (23.6%)	25 (25.8%)	
Reason for admission					
Rehabilitation	86 (36.1%)	26 (30.2%)	24 (43.6%)	36 (37.1%)	0.001
Skilled-nursing care	49 (20.6%)	7 (8.1%)	15 (27.3%)	27 (27.8%)	
Intermediate care	8 (3.4%)	4 (4.7%)	1 (1.8%)	3 (3.1%)	
Respite	95 (39.9%)	49 (57.0%)	15 (27.3%)	31 (32.0%)	
Place veterans came from					
Home	101 (42.4%)	54 (62.8%)	15 (27.3%)	32 (33.0%)	<0.001
Acute care	128 (53.8%)	28 (32.6%)	38 (69.1%)	62 (63.9%)	
Other nursing home	9 (3.8%)	4 (4.7%)	2 (3.6%)	3 (3.1%)	
Season of admission					
Winter	62 (26.1%)	23 (37.1%)	13 (21.0%)	26 (41.9%)	0.83
Spring	55 (23.1%)	18 (32.7%)	16 (29.1%)	21 (38.2%)	
Summer	62 (26.1%)	20 (32.3%)	14 (22.6%)	28 (45.2%)	
Autumn	59 (24.8%)	25 (42.4%)	12 (20.3%)	22 (37.3%)	
Correlation between FI and 25 (OH) D^a^	−0.10 *p* = 0.13	−0.05 *p* = 0.63	−0.19 *p* = 0.02		
Relative risk of frailty, OR (95% CI)	1.0 (ref)	1.12 (1.05–1.18) *p* < 0.001	1.04 (0.94–1.15) *p* = 0.42	0.87 (0.77–0.99) *p* = 0.03	

Analysis of variance for continuous variables and chi-square test for categorical variables mean ± Standard deviation, n (%)

*25 (OH) D* 25-hydroxyvitamin D level, *95%CI* 95% confidence interval, *FI* Frailty Index, *OR* Odds ratio

^a^Spearman’s correlation coefficient in supplement users and non-users

The characteristics were compared across three groups by CART analysis: supplement users (*n* = 86, 36.1%), non-users with low vitamin D (*n* = 55, 23.1%), and non-users with high vitamin D (*n* = 97, 40.8%) (Table 1). The mean FI and prevalence of frailty were both significantly different across the groups, with supplement users being the most frail and non-users with high vitamin D being the least frail (mean FI: 0.43, 0.41, and 0.35, respectively. Frailty prevalence: 97.7, 90.9, and 76.3%, respectively. Both *p* < 0.001). Supplement users and non-users with high vitamin D had significantly higher 25 (OH) D, 26.6 and 27.1 ng/mL, respectively, than non-users with low vitamin D, 11.7 ng/mL. While FI and 25 (OH) D were not significantly correlated in the entire cohort and supplement users (*r* = −0.10 and −0.05, respectively), there was a significant inverse correlation between FI and 25 (OH) D in non-users (*r* = −0.19, *p* = 0.02).

Table 2 shows univariate and age-adjusted logistic regression models used to assess risk of frailty (FI ≥ 0.25). In unadjusted models, supplement users and non-users with low vitamin D were significantly more likely to be frail (Odds ratio [OR] = 13.05, 95% CI = 2.98–57.25, *p* = 0.001; OR = 3.11, 95% CI = 1.11–8.72, *p* = 0.03, respectively), with non-users with high vitamin D as a reference. In age-adjusted models, the risk of frailty for supplement users and non-users with low vitamin D remained significant after adjusting for age (OR = 9.90, 95% CI = 2.18–44.86, *p* = 0.003; OR = 4.28, 95% CI = 1.44–12.68, *p* = 0.009, respectively).

**Table 2:** Univariate and age-adjusted logistic regression models for frailty

	Unadjusted		Age-adjusted	
Factors	Odds Ratio (95% CI)	*p* value	Odds Ratio (95% CI)	*p* value
Three groups by CART analysis				
Non-user with high 25 (OH) D	1.0 (ref)	–	1.0 (ref)	–
Non-user with low 25 (OH) D	3.11 (1.11–8.72)	0.03	4.28 (1.44–12.68)	0.009
Supplement user	13.05 (2.98–57.25)	0.001	9.90 (2.18–44.86)	0.003
25 (OH) D (ng/mL)	0.98 (0.95–1.02)	0.39	–	–
Age (years)	1.06 (1.03–1.10)	<0.001	–	–
Body mass index	1.01 (0.95–1.07)	0.84	–	–
Education (year)	0.90 (0.76–1.08)	0.26	–	–
Ethnicity				
White	1.0 (ref)	–	–	–
Asian/PI	2.13 (0.92–4.92)	0.08	–	–
Others	3.51 (0.44–27.87)	0.24	–	–
Smoking				
Never	1.0 (ref)	–	–	–
Past	1.59 (0.68–3.74)	0.29	–	–
Current	0.94 (0.32–2.73)	0.91	–	–
Alcohol				
Never	1.0 (ref)	–	–	–
Past	1.12 (0.45–2.81)	0.81	–	–
Current	0.70 (0.27–1.78)	0.45	–	–

*25 (OH) D* 25-hydroxyvitamin D level, *95% CI* 95% confidence interval, *CART* Classification and regression tree

**Fig. 2:**
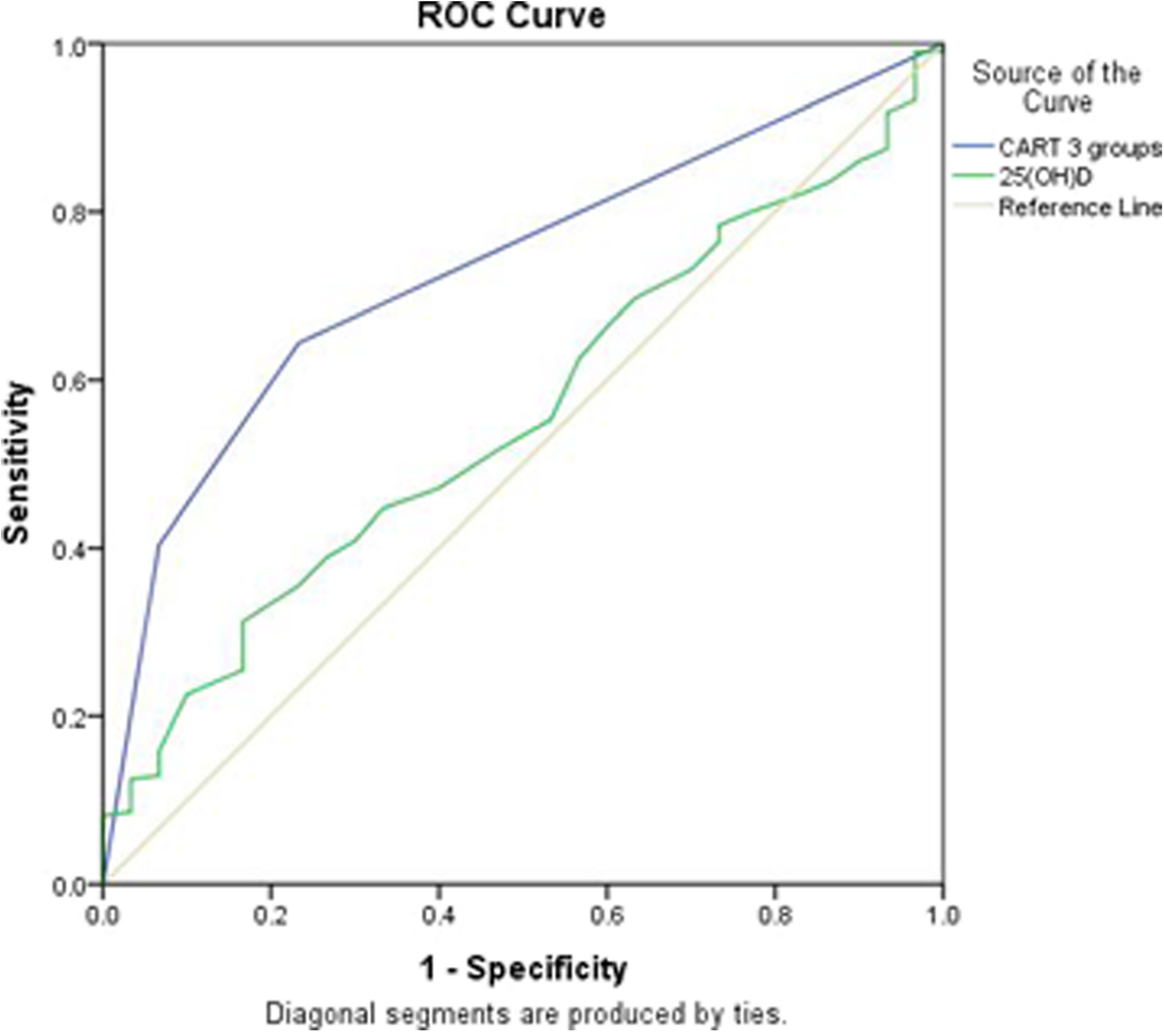
Receiver operating characteristic (ROC) curve analyses for risk of frailty predicted by the classification and regression tree (CART) analysis groups (blue line) and 25-hydroxyvitamin D (25 (OH) D) level (green line). 25 (OH) D: Serum total 25-hydroxyvitamin D. CART: Classification and regression tree. Area under the ROC curve (AUC) = 0.73 (95% confidence interval (CI) = 0.65–0.82, *p* < 0.001) for CART analysis groups (blue line); AUC = 0.56 (95%CI = 0.46–0.66, *p* = 0.32) for 25 (OH) D (green line)

Figure 2 shows an ROC curve analysis showing that CART analysis groups accurately classified risk of frailty among the entire cohort, with AUC of 0.73 (95%CI = 0.65–0.82, *p* < 0.001). Being on a vitamin D supplement, compared with no use, has sensitivity of 64.4% and specificity of 76.7%. Conversely, 25 (OH) D was not a significant predictor of frailty (AUC = 0.56 95% CI = 0.46–0.66, *p* = 0.32). After removing supplement users in order to exclude effects of vitamin D supplementation, ROC curve analysis for 25 (OH) D was repeated only among non-users (*n* = 152). This repeated analysis showed that 25 (OH) D significantly predicted frailty among non-users, with AUC of 0.62 (95% CI = 0.52–0.73, *p* = 0.04) (Figure not shown).

Among 86 supplement users, the dosages of vitamin D supplementation were 1–400 IU/day (*n* = 27, 31.4%), 401–800 IU/day (*n* = 38, 44.2%), and ≥801 IU/day (*n* = 21, 25.3%). Table 3 compares the three groups and shows that higher dosage of vitamin D supplementation was significantly associated with higher 25 (OH) D (p for trend = 0.003) and higher body mass index (*p* = 0.05). Although statistical significance was not reached, those on higher dosage of supplementation were more likely from home and less likely from acute care hospitals (*p* = 0.06).

**Table 3:** Characteristics of vitamin D supplement users according to the dosage

	*N* = 86	1–400 IU/day (*n* = 27)	401–800 IU/day (*n* = 38)	801+ IU/day (*n* = 21)	*p* value
Frailty Index	0.43 ± 0.11	0.45 ± 0.11	0.40 ± 0.09	0.43 ± 0.12	0.59
Frailty (FI > =0.25)	84 (97.7%)	25 (92.6%)	38 (100.0%)	21 (100.0%)	0.11
25 (OH) D (ng/mL)	26.6 ± 9.3	22.9 ± 8.6	26.8 ± 8.4	30.9 ± 10.2	0.003
Vitamin D deficiency	19 (22.1%)	9 (33.3%)	7 (18.4%)	3 (14.3%)	0.10
Age	79.0 ± 11.7	79.7 ± 9.5	80.9 ± 11.1	74.7 ± 14.6	0.17
Body mass index	25.1 ± 6.0	23.9 ± 4.7	24.7 ± 5.8	27.5 ± 7.5	0.05
Education (year)	13.0 ± 2.4	13.1 ± 1.8	12.7 ± 2.7	13.4 ± 2.7	0.72
Ethnicity					
White	41 (47.7%)	13 (48.1%)	22 (59.5%)	6 (28.6%)	0.28
Asian/PI	41 (47.7%)	13 (48.1%)	14 (37.8%)	14 (66.7%)	
Others	3 (3.5%)	1 (3.7%)	1 (2.7%)	1 (4.8%)	
Reason for admission					
Rehabilitation	26 (30.2%)	13 (48.1%)	8 (21.1%)	5 (23.8%)	0.11
Skilled-nursing care	7 (8.1%)	0 (0.0%)	5 (13.2%)	2 (9.5%)	
Intermediate care	4 (4.7%)	2 (7.4%)	2 (5.3%)	0 (0.0%)	
Respite	49 (57.0%)	12 (44.4%)	23 (60.5%)	14 (66.7%)	
Place veterans came from					
Home	54 (62.8%)	12 (44.4%)	26 (68.4%)	16 (76.2%)	0.06
Acute care	28 (32.6%)	14 (51.9%)	11 (28.9%)	3 (14.3%)	
other nursing home	4 (4.7%)	1 (3.7%)	1 (2.6%)	2 (9.5%)	
Smoking					
Never	27 (31.4%)	6 (22.2%)	14 (36.8%)	7 (33.3%)	0.75
Past	49 (57.0%)	18 (66.7%)	20 (52.6%)	11 (57.0%)	
Current	10 (11.6%)	3 (11.1%)	4 (10.5%)	3 (14.3%)	
Alcohol					
Never	39 (45.3%)	11 (40.7%)	18 (47.4%)	10 (47.6%)	0.82
Past	30 (34.9%)	11 (40.7%)	11 (28.9%)	8 (38.1%)	
Current	17 (19.8%)	5 (18.5%)	9 (23.7%)	3 (14.3%)	

One-way ANOVA for continuous variables (p for trend) and chi-square test for categorical variables. mean ± standard deviation, n (%)

## Discussion

In the current study, three distinct subgroups were successfully identified based on use of vitamin D supplementation and 25 (OH) D using the CART analysis. Taking vitamin D supplementation was the strongest frailty risk discriminative factor, and supplement users were found to have multiple distinguishable features. They were almost all frail, with the highest prevalence of 97.7% (84/86) among the three subgroups, but had high mean 25 (OH) D of 26.6 ng/mL, which was compatible with non-users with high vitamin D (27.1 ng/mL). Among supplement users, 25 (OH) D was not correlated with FI, while there was a significant correlation among non-users.

In light of these findings, we speculate that vitamin D supplement users are a group of highly frail older people with paradoxically high 25 (OH) D levels. This discrepancy between 25 (OH) D and frailty may result from the fact that vitamin D supplementation can quickly correct low 25 (OH) D [40]; however, it may take longer to see positive effects on mortality or other outcomes [11, 41]. One meta-analysis showed that vitamin D supplementation significantly decreased mortality only with follow-up longer than 3 years [41]. In addition, supplements increase 25 (OH) D more efficiently in those with lower baseline 25 (OH) D, [42] which will further exacerbate the discrepancy. Therefore, frail supplement users are likely to remain at high risk of negative health outcomes even with optimized vitamin D status by supplementation, which could potentially confound the profiles and outcomes of the vitamin D-sufficient group. In observational studies, true associations of 25 (OH) D with outcomes could be attenuated or reversed at high 25 (OH) D levels, creating falsely null or U-shaped associations unless controlled properly for the use of vitamin D supplements.

Vitamin D supplements have been used more and more commonly in general populations. According to NHANES, the use of dietary vitamin D supplements has increased among both men and women in most age groups from 1988 to 1994 to 2003–2006 [43]. This increase may be explained by several reasons. With guidelines and authorities advocating the importance of treating low vitamin D, clinicians more often check patients’ serum vitamin D levels and prescribe vitamin D supplement for low vitamin D. Increasing sun protection for skin cancer prevention can also contribute to the increase in vitamin D supplementation. Due to growing media exposure regarding risks of low vitamin D levels, the general public has more awareness of the importance of vitamin D than ever. Furthermore, vitamin D supplements are inexpensive and readily available over the counter with almost no serious adverse effects, which further lowers the threshold for people to start the supplements. In this context, future vitamin D research should take into account the possibility of high prevalence of vitamin D supplementation in various populations, which may have significant confounding effects.

This study has some limitations and its findings must be interpreted with caution. First, it included a small number of only male veterans in a nursing home at a single facility in Hawaii, and the findings may not be entirely generalizable to other populations or to women. Second, some important information related to vitamin D status is missing, such as dietary vitamin D intake, sunlight exposure, or reasons for vitamin D supplementation. Third, we did not have information on what assay was used to measure 25 (OH) D given that 25 (OH) D levels vary according to the assay used [44]. Fourth, due to the small number of participants, we included those who had available data for at least 70% of the deficits, instead of 80%, which is typically required for calculation of the FI [36]. Fifth, the multiple imputation could have been conducted for missing value of 25 (OH) D. Lastly, the cross-sectional study design hinders assessing prospective associations and interactions among 25 (OH) D, vitamin D supplementation, and health outcomes, as vitamin D supplementation may improve supplement users’ overall health status and decrease the risks over years.

## Conclusion

Three distinct groups were categorized from the study: 1) vitamin D supplement users, 2) non-users with low vitamin D, and 3) non-users with high vitamin D levels. This study shows that use of vitamin D supplements can potentially be a cause of paradoxical U-shaped associations between vitamin D levels and negative health outcomes, by creating a unique group of participants who are the most frail but have high 25 (OH) D levels. This highlights the importance of identifying vitamin D supplement users and ideally obtaining information on the dosage and duration of the supplementation in order to better examine the true association between vitamin D and health outcomes, including frailty status, by controlling for vitamin D supplementation effects. Future studies can further examine and clarify these effects.

## Additional file

Additional file 1: List of 34 deficits for constructing Frailty Index. (DOC 90 kb)

## Abbreviations

25 (OH) D: 25-hydroxyvitamin D
ANOVA: Analysis of variance
CART: Classification and regression tree
FI: Frailty Index
ROC: Receiver operating characteristic
