# Supplementary material for: Vitamin D supplementation as a potential cause of U-shaped associations between vitamin D levels and negative health outcomes: a decision tree analysis for risk of frailty
Source: BMC Geriatr. 2017 Oct 16;17:236. doi: 10.1186/s12877-017-0631-0 (PMC5644251; doi:10.1186/s12877-017-0631-0)
Supplement: Additional file 1: — List of 34 deficits for constructing Frailty Index. (DOC 90 kb) [file 12877_2017_631_MOESM1_ESM.doc]

**Appendix.** List of 34 deficits for constructing Frailty Index

|  | variable | grading |
| --- | --- | --- |
|  | **Chronic diseases (n=12)** |  |
| 1 | Hypertension | Present=1, absent=0 |
| 2 | Diabetes mellitus | Present=1, absent=0 |
| 3 | Hyperlipidemia | Present=1, absent=0 |
| 4 | Coronary artery disease | Present=1, absent=0 |
| 5 | Cerebrovascular accident | Present=1, absent=0 |
| 6 | Cancer | Present=1, absent=0 |
| 7 | Depression | Present=1, absent=0 |
| 8 | Dementia | Present=1, absent=0 |
| 9 | Kidney disease | Present=1, absent=0 |
| 10 | Liver disease | Present=1, absent=0 |
| 11 | Osteoporosis | Present=1, absent=0 |
| 12 | Amputation | Present=1, absent=0 |
|  | **Psychological symptoms (n=9)** |  |
| 13 | Feeling hopeless | Yes=1, No=0 |
| 14 | Feeling worthless | Yes=1, No=0 |
| 15 | Feeling happy | Yes=0, No=1 |
| 16 | Feeling helpless | Yes=1, No=0 |
| 17 | Being in good spirits | Yes=0, No=1 |
| 18 | Often getting bored | Yes=1, No=0 |
| 19 | Having dropped activities and interests | Yes=1, No=0 |
| 20 | Being satisfied with life | Yes=0, No=1 |
| 21 | Feeling full of energy | Yes=0, No=1 |
|  | **Functional disabilities (n=6)** |  |
| 22 | Difficulty with mobility | Present=1, absent=0 |
| 23 | Difficulty with bathing | Present=1, absent=0 |
| 24 | Difficulty with dressing | Present=1, absent=0 |
| 25 | Difficulty with toileting | Present=1, absent=0 |
| 26 | Difficulty with being incontinent | Present=1, absent=0 |
| 27 | Difficulty with feeding | Present=1, absent=0 |
|  | **Gait/fall-related problems (n=3)** |  |
| 28 | History of fall | Present=1, absent=0 |
| 29 | Walking aid | Present=1, absent=0 |
| 30 | Gait problem | Present=1, absent=0 |
|  | **Cognitive symptoms (n=2)** |  |
| 31 | Orientation problem | Present=1, absent=0 |
| 32 | Memory problem | Present=1, absent=0 |
| 33 | **Obesity (n=1)** | BMI>30=1, BMI<30=0 |
| 34 | **Pain symptoms (n=1)** | Present=1, absent=0 |
